# Breaking the Cycle: Why Women of Reproductive Age Belong in All Areas of Health Research

**DOI:** 10.1111/1471-0528.18255

**Published:** 2025-06-16

**Authors:** Rachel Hulme, Margherita Bielli, Kate Womersley, Edward Mullins

**Affiliations:** ^1^ The George Institute for Global Health UK; ^2^ Department of Metabolism, Digestion and Reproduction Imperial College London UK; ^3^ Imperial College London The George Institute for Global Health UK; ^4^ Imperial College London The George Institute for Global Health, Imperial College Healthcare NHS Trust UK

**Keywords:** epidemiology: perinatal, risk management, translational research

Medical research and clinical practice have a longstanding sex and gender problem. There is, and has historically been, an over‐representation of male participants in biomedical research: only 20% of participants in phase 1 clinical trials are women, while up to 5.5 times more male than female cells and animals are included in preclinical research [1, 2]. It is estimated that only 5%–14% of studies across disciplines analyse outcomes by sex [3]. Sex and gender bias in research reinforces inequities in medical knowledge and clinical care. These issues of under‐representation are further compounded for women who also belong to other marginalised groups due to their race, ethnicity, socio‐economic status, or as a result of chronic health conditions, as seen, for example, in racial disparities in prescription practices, particularly in pain management and comorbidity treatment [4]. This commentary addresses two intersecting but distinct patterns: the exclusion of pregnant women, and women of perceived childbearing potential from biomedical research.

The scale and impact of women's under‐representation continue to be exposed. Efforts to redress this inequality have been led by specialities such as cardiology, through work on cardiomyopathy fatalities to abdominal aortic aneurysm repair outcomes [5, 6]. If more specialities were committed to discovering inequalities in their practices, many more examples of such inequality would be found hiding in plain sight. At least 56% of disability‐adjusted life years (DALYs) stem from conditions which impact women differently or disproportionately to men. Beyond the damaging, sometimes life‐threatening, impacts on women's health, exclusion of women from biomedical research is also economically harmful. The management consultancy McKinsey recently modelled that closing the global women's health gap would generate a $1 trillion annual saving by 2040, and in the UK alone this would amount to $36 billion per annum by 2040 (Data commissioned and presented by Dame Lesley Regan, Women's Health Ambassador for England, on 7/4/25 at The 7th Venice Forum conference in Florence).

In the UK, the MESSAGE project (Medical Science Sex and Gender Equity), hosted at the George Institute for Global Health, Imperial College London and funded by Wellcome, has intervened to improve well‐described sex and gender inequalities in research inclusion, analysis and reporting [7]. From 2022 to 2024, MESSAGE hosted a series of cross‐sector policy labs to co‐design a sex and gender policy framework for research funders in the UK [8].

Together with representatives from across the research sector, including patients, funders, regulators, clinicians, researchers and academic publishers, MESSAGE generated a workable consensus to bring UK clinical research to a world‐leading position of inclusion for the benefit of everyone in society. In 2024, MESSAGE publicly launched its landmark sex and gender policy framework for UK research funders, which sets a new gold standard for scientific consideration of sex and gender in biomedical, health and care research. Throughout, the MESSAGE project has consistently emphasised the adoption of a lifecourse approach to women's health, aligning with frameworks established by the Royal College of Obstetricians and Gynaecologists (RCOG) and the Department of Health and Social Care's (DHSC) 2022 Women's Health Strategy.

MESSAGE has underscored the necessity of broadening the conceptualisation of women's health beyond the traditional parameters of reproductive health. This includes addressing sex and gender inequities across the gamut of medical specialities, rather than maintaining an exclusive or predominant focus on obstetrics and gynaecology. Encouragingly, the MESSAGE framework is already being widely adopted by key funders across the sector, from national organisations such as the National Institute of Health and Care Research (NIHR) to smaller charitable funders driving crucial work on single disease areas, such as Fight for Sight and Parkinson's UK.

During its cross‐sector consultations, the MESSAGE project team has repeatedly heard rationales attempting to explain the continued exclusion of women from clinical trials, which could yield potentially beneficial interventions for lifelong health. These attempted justifications arise from viewing women through the lens of their reproductive capacity.

Firstly, stakeholders voiced concerns that women's perceived complexity will ‘muddy’ research findings due to the ‘disruptive’ effect of the menstrual cycle and problematised hormonal variation across the lifecourse from menarche to menopause. This overlooks the importance of understanding the effects that hormones, menstruation and reproductive events have on women's general health in favour of excluding them for the sake of ‘cleaner’ data.

Secondly, there is a fear of teratogenicity and fetal harm in cases of potential pregnancy. Researchers voiced a pervasive worry that females from 10 to 55 years of age are at an unconscionable risk of conceiving, even if they are using contraception. It is well known that fear of teratogenesis since the harm of thalidomide in the early 1960s has driven a significant proportion of women's exclusion from clinical research, with a lasting legacy still seen in women's participation and inclusion across all areas of medical research.

Thirdly, researchers are typically deterred by the significant burden and expense of meeting regulatory and ethical standards for trials that include women who could become pregnant. This is particularly problematic for small‐scale studies where no plausible fetal harm could occur; nevertheless, researchers face obstacles if they seek to include pregnant women. For the pharmaceutical industry especially, companies see significant reputational and financial risks in challenging the status quo when the precautionary principle is a well‐established norm [9].

The decades‐long systematic under‐representation of pregnant women in clinical trials has led to their ‘therapeutic orphan’ status [10]. This term highlights the substantial knowledge gap regarding the safe use of medications during pregnancy. Twenty per cent of women enter pregnancy with a pre‐existing medical condition for which they may take medication, and most women use at least one prescribed medicine during pregnancy. Yet over 90% of all medications lack sufficient safety data for confident use during the perinatal period. As a result, a large majority of medications are prescribed ‘off‐label’—beyond their approved therapeutic scope—placing both the health of the mother and the fetus at risk.

This reflects broader structural issues within medical research that systematically deprioritise the inclusion of women and gender‐diverse people. One relevant factor is the predominance of men as Principal Investigators (PIs), which is known to influence perceptions of urgency and prioritisation of sex and gender diversity in study design and implementation [11]. The under‐representation of women in leadership positions not only skews the research agenda but also reinforces existing gender biases in scientific inquiry. Further, evidence highlights that gender imbalances extend beyond PIs to include investigators, journal editors and peer reviewers, whose collective influence shapes research priorities, funding allocations and the publication landscape [12]. For instance, studies have shown that editorial boards dominated by men are less likely to prioritise research on women's health, while reviewers who are men are more likely to exhibit implicit biases that devalue or reject such studies. These structural barriers perpetuate a feedback loop of under‐representation, diminishing both the visibility and urgency of addressing women's health needs. Tackling these systemic biases requires deliberate interventions, such as improving gender diversity among key decision‐makers in research and instituting policies that prioritise sex‐ and gender‐sensitive research to promote equity across all stages of the clinical research pipeline.

The prevailing norms of excluding women from clinical research, particularly justified by pregnancy‐related risks, are often presented to researchers, clinicians and society as prudent and appropriately protective. This rationale is largely based on a narrow conception of risk, concerned with measurable but rare fetal outcomes (particularly malformations), while under‐emphasising the immediate and long‐term risks to maternal health posed by evidence gaps. The prioritisation of hypothetical fetal risks over more immediate maternal risks—such as those arising from poorly managed pre‐existing conditions or perinatal complications—reflects a partial understanding of the maternal‐fetal dyad [13]. To divide risks into maternal versus fetal overlooks the significance of their shared wellbeing. Avoiding interventions and medications due to often theoretical risks of foetal abnormalities may risk significantly destabilising a mother's health, in turn destabilising the environment in which the fetus is developing.

Increasing recognition of the need to account for the risks of the precautionary principle, to weigh, for example, potential adverse neonatal effects of mood stabiliser exposure against the benefit of stabilised maternal mental health, has begun to shift this perspective. However, there remains a tendency to focus exclusively on risks for the small numbers of women enrolled in research and clinical trials, neglecting the far broader risks which are imposed at scale on women who, in the absence of sufficient evidence, are forced to make healthcare decisions which are under‐informed by data. The larger scale implications of research exclusion, where the omission of women ultimately compromises the quality of care available to pregnant women and those of childbearing age, generate an ongoing cohort effect and cycle of exclusion which entrenches the health inequalities faced by women [14, 15, 16].

The lack of improvement regarding the inclusion of pregnant women in clinical research since the 1960s is partly due to a fragmented distribution of responsibility. Different stakeholder groups—such as pharmaceutical companies, researchers, funders and policymakers—deflect responsibility to one another or insist that another stakeholder group must be the ‘first mover’ to address such challenges. This is understandable, but unsustainable. When starting MESSAGE we observed this dynamic which had been stymying coordinated progress, with each group—funders, universities, regulators, journals—assuming that others were in a stronger position to take the lead.

The solution lies in cross‐sector, synchronised action, reframing the question of inclusion as an opportunity for collaboration rather than censure. Efforts should draw on expertise from fields beyond biomedical research and epidemiology to welcome perspectives from bioethics, legal studies, policy groups and patient voices to refine clinical trial design, risk assessment and regulatory frameworks while centring women's autonomy and agency.

Pharmaceutical companies, for example, must be engaged as collaborators rather than blamed as self‐servingly hesitant about including women of reproductive age in research to find new strategies that balance safety with the need for evidence when treating pregnant and postpartum women. Integrating the perspectives of individuals with lived experience of pregnancy, birth and related complications is essential to ensure research is relevant and valuable to the priorities of women and their families. Emphasising intersectionality in this process must not be an afterthought, especially given the shameful disparities in maternal outcomes of Black and South Asian women giving birth in the UK and US. The experiences of women from diverse backgrounds—including those of different racial, ethnic, socio‐economic and health status groups—must be meaningfully considered to ensure that research is truly inclusive and responsive to the varied needs of all women, particularly those from marginalised communities.

Addressing the systemic exclusion of women from clinical research, particularly due to concerns surrounding pregnancy‐related risks, demands a paradigm shift in research methodology and structure [13]. Prioritising the autonomy and health needs of women over academic or institutional norms is long overdue. The focus must transition from theoretical discourse and ‘admiring the problem’, to impact‐driven coordinated change, with an emphasis on influencing policy and driving practical improvements in research practice that robustly account for the harms of exclusion rather than focusing on the risks of inclusion. Efforts to advance sex and gender equity in research must also contend with broader political headwinds, including the erosion of reproductive rights and ideological opposition to initiatives promoting research inclusion in certain countries across the globe. Given these contexts, transparent communication, public education and strong alliances across science, policy and civil society will be vital to sustaining progress.

## Author Contributions

K.W., E.M., R.H. and M.B. conceived of this commentary as a group. R.H. and K.W. wrote the first draft, which was expanded and revised by all authors. All authors have approved the final version and accept responsibility for the paper as published.

## Ethics Statement

The authors have nothing to report.

## Conflicts of Interest

K.W. is co‐principal investigator of the MESSAGE project which has been funded by Wellcome.

## Data Availability

Data sharing is not applicable to this article as no new data were created or analyzed in this study.
